# Comparative Performance of Large Language Models in Muscle Histology Classification Highlights Enhanced Accuracy of ChatGPT-4o in Tissue Identification

**DOI:** 10.7759/cureus.90103

**Published:** 2025-08-14

**Authors:** Parth Shah, David J Boughanem, John M Templeton, Marzenna Wiranowska, Karim Hanna

**Affiliations:** 1 Medicine, University of South Florida Morsani College of Medicine, Tampa, USA; 2 Computer Science and Engineering, Bellini College of Artificial Intelligence, Cybersecurity, and Computing, University of South Florida, Tampa, USA; 3 Medical Engineering, University of South Florida College of Engineering, Tampa, USA; 4 Pathology and Cell Biology, University of South Florida Morsani College of Medicine, Tampa, USA; 5 Family Medicine, University of South Florida Morsani College of Medicine, Tampa, USA

**Keywords:** artificial intelligence (ai), artificial intelligence (ai) in medicine, artificial intelligence in health care, digital pathology, histology and histopathology, multimodal large language model, muscle tissue

## Abstract

Introduction

One of the most promising avenues of artificial intelligence (AI) integration into medicine is its examination, evaluation, and characterization of pathological slides. The use of large language models (LLMs), the AI model subtype that is becoming increasingly popular, in pathological applications remains unexplored. This study investigates the histological image recognition capabilities of the multimodal models Gemini 1.5 Flash, ChatGPT-4o, and Claude 3.5 Sonnet and assesses their suitability for clinical or medical education use.

Methods

The models were evaluated using 300 digital histology images derived from the University of South Florida Morsani College of Medicine Virtual Microscopy database, with a prompt to ascertain each model's ability to identify tissue type and plane of sectioning used. The images included the three subtypes in both longitudinal and transverse planes of sectioning. Standard machine learning metrics such as precision, recall, accuracy, and F1 score were used to classify and evaluate each model’s abilities.

Results

In the prediction of tissue type, OpenAI’s ChatGPT had the highest metrics with an F1 score of 0.772, while Claude yielded an F1 score of 0.380, and Gemini produced a 0.460 F1 score. In the prediction of sectioning, ChatGPT produced an F1 score of 0.396, while Claude produced a value of 0.472, and Gemini yielded 0.344.

Conclusion

Overall, the results indicate that ChatGPT is most effective at identifying tissues. However, the inaccuracy demonstrated in evaluating sectioning compared to other models leaves room for improvement in its overall accuracy across varying tissue samples to reliably supplement medical education or clinical use.

## Introduction

The integration of artificial intelligence (AI) into medicine is a watershed moment and has the potential to transform the healthcare industry [1,2]. In particular, the application of AI in fields that use a large volume of imaging technology has the potential to bring substantial efficiency and workflow improvements and enhance the speed and accuracy of diagnoses [2,3]. One such field is diagnostic pathology, where the increasing use of whole-slide imaging technologies and FDA approval of these techniques for primary diagnoses have created an opportunity to apply AI to provide workflow improvements and help triage diagnoses, providing valuable decision-making support to pathologists and potentially reducing backlog [4-6]. 

Large language models (LLMs), a type of machine learning model based on a transformer architecture that can process information in parallel, demonstrate greater scalability and computational efficiency for complex tasks [7]. These models have been developed to include multimodal capabilities and thus interpret visual and auditory inputs. Although image classification tasks have historically used convolutional neural networks (CNNs), vision transformers (ViT) use a similar underlying transformer model as LLMs can perform better than CNN-based systems in certain image classification tasks [7,8]. In addition, these multimodal models possess zero-shot or few-shot learning capabilities, which allow them to adapt to answer a wide range of queries without explicit training [7]. The clinical potential and benefits of LLM integration in medical applications have been investigated, and the technology has demonstrated the potential to assist with medical records and healthcare delivery, as well as the interpretation of clinical information and applications in medical education [9-13]. In addition, the visual interpretation abilities of multimodal LLMs have also been investigated, and studies have demonstrated their potential clinical application in interpreting 12-lead EKGs [14,15].

The clinical usefulness of AI in pathological applications is dependent on the ability of the tool to identify histological features and differentiate between tissue types. Prior studies have found that AI models can be useful in histopathology applications, specifically for making prostate cancer diagnoses [16]. The use and performance of publicly available general-purpose LLMs in histopathological applications remain largely unexplored. Evaluating the ability of these rapidly evolving models to pinpoint histological structures and patterns and identify tissue types is vital in determining the feasibility of integrating these tools or derivatives of these tools into digital pathology workflows. This study evaluates the histological classification abilities of three multimodal LLMs: ChatGPT-4o (OpenAI, San Francisco, CA, USA), Gemini 1.5 Flash (Google LLC, Mountain View, CA, USA), and Claude 3.5 Sonnet (Anthropic, San Francisco, CA, USA). These models were chosen for their wide availability and use, and rapid rate of development. Specifically, the performance of each of the models in differentiating between the three major muscle subtypes (smooth, skeletal, and cardiac) and identifying the plane of sectioning used in each image was assessed. Developing an understanding of the ability of each of these untrained models’ capabilities in these fundamental competencies provides a gauge of their readiness for clinical or educational pathology applications.

This article was previously presented as a poster at the 2025 University of South Florida Annual Research Day on February 28, 2025, and was submitted as a poster for the Florida Medical Association Annual Meeting on July 26, 2025.

## Materials and methods

Three of the most popular multimodal LLMs (OpenAI’s ChatGPT-4o, Google’s Gemini 1.5 Flash, and Anthropic’s Claude 3.5 Sonnet) were selected as the models to be assessed for their ability to distinguish between images of normal muscle histology. These versions of each model were the most up-to-date and freely available at the time of data collection (December 2024). The presorted histology images used were acquired from the University of South Florida (USF) Morsani College of Medicine (Tampa, FL, USA) virtual microscopy database created by the USF Information Technology Advanced Visualization Center. Institutional permission was obtained for the use of university materials for this project.

**Figure 1:**
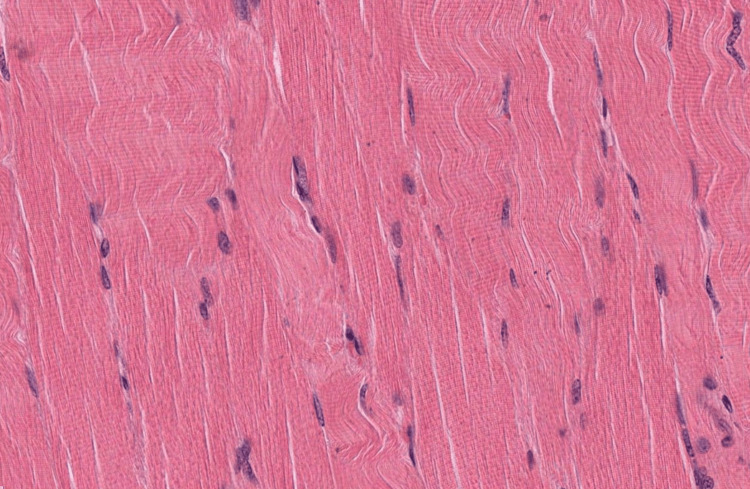
An image of the longitudinal section of skeletal muscle tissue that was uploaded into each of the LLMs LLM: Large language model

**Figure 2:**
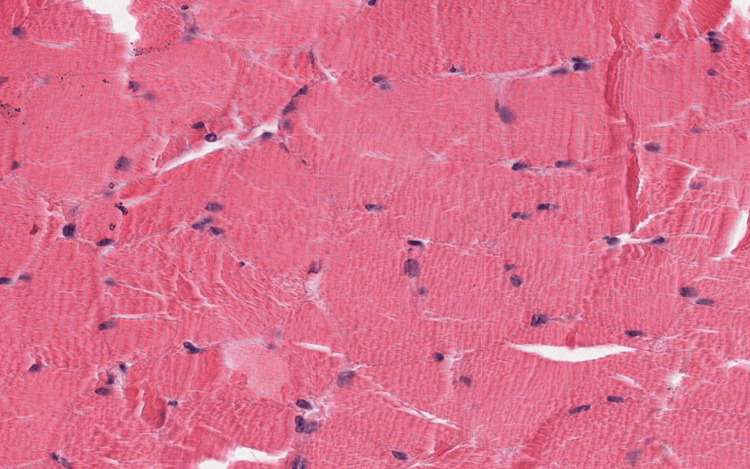
An image of the cross-section of skeletal muscle tissue that was uploaded into each of the LLMs LLM: Large language model

**Figure 3:**
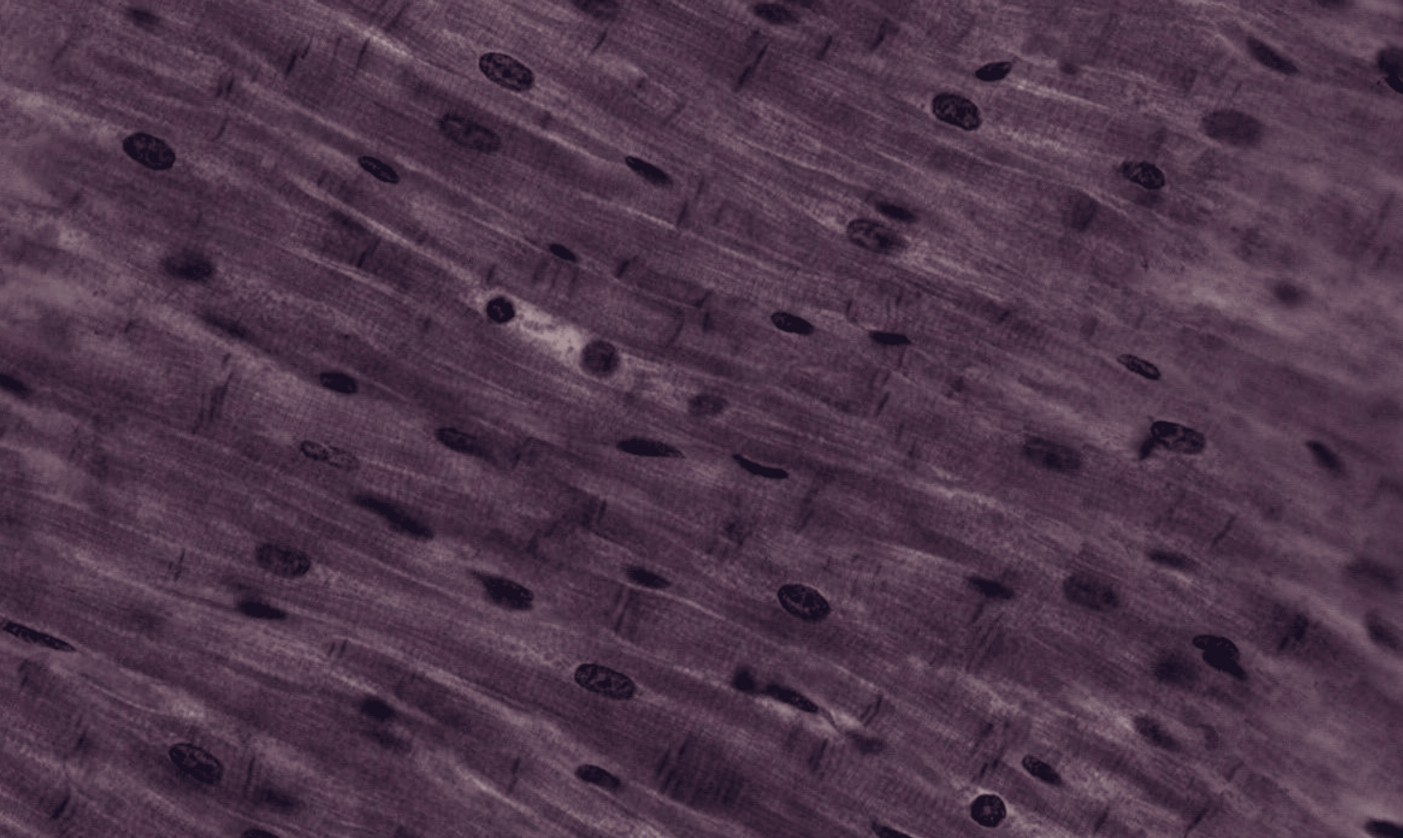
An image of the longitudinal section of cardiac muscle tissue that was uploaded into each of the LLMs LLM: Large language model

**Figure 4:**
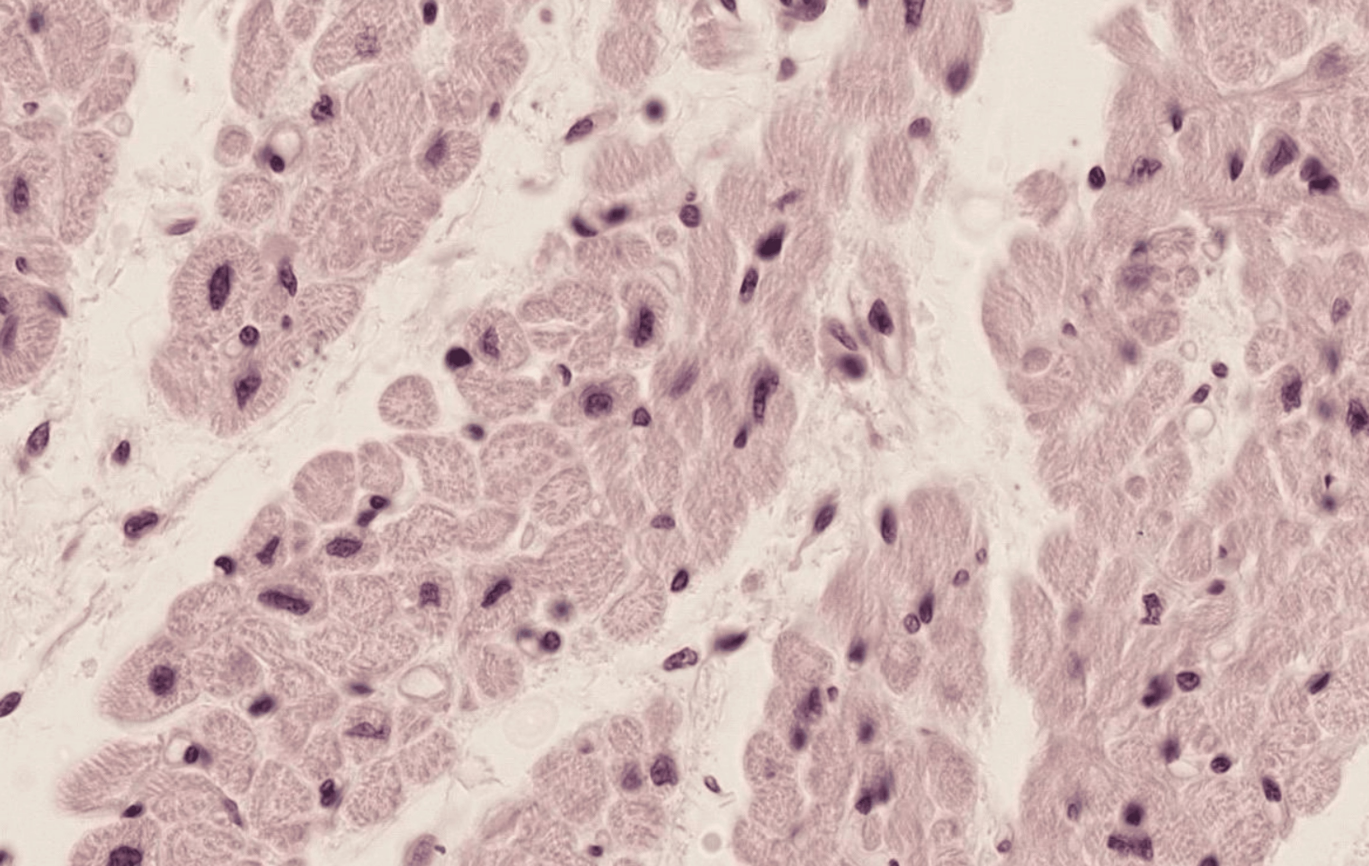
An image of the cross-section of cardiac muscle tissue that was uploaded into each of the LLMs LLM: Large language model

**Figure 5:**
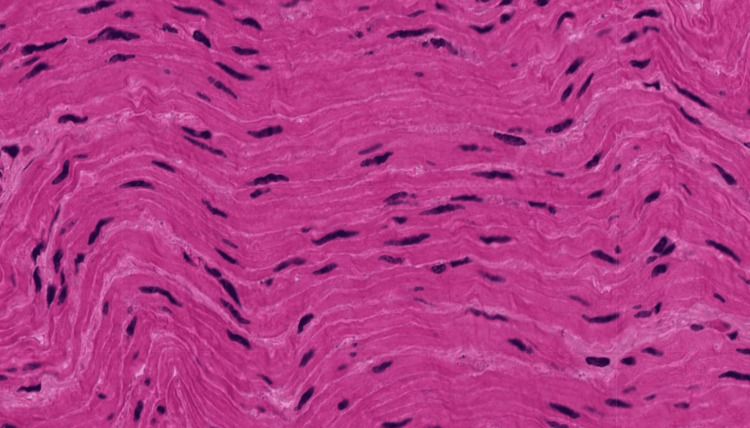
An image of the longitudinal smooth muscle tissue that was uploaded into each of the LLMs LLM: Large language model

**Figure 6:**
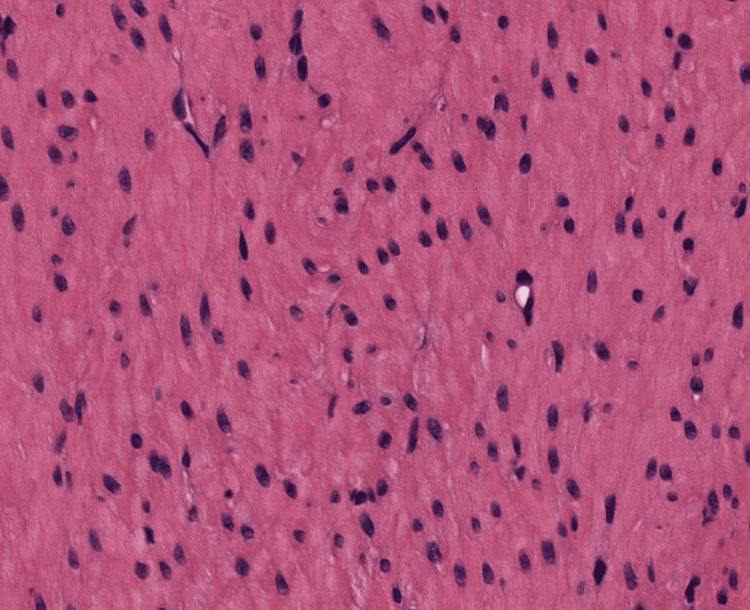
An image of the cross-section of smooth muscle tissue that was uploaded into each of the LLMs LLM: Large language model

To maintain the diversity of data given practical constraints, a total of 300 images were extracted from the database, stratified equally into skeletal, smooth, and cardiac muscle. Each image primarily featured tissue characteristic of a specific muscle subtype but may have also included other muscle fiber-associated tissue, such as connective tissue and blood vessels. Each set of 100 images per muscle subtype was further split between 50 images in a cross-sectional plane and 50 images in a longitudinal plane. For each image, the highest available magnification (100× or 200×, beyond the original 40× image capture) was applied, focusing on small regions of the slide measuring approximately 10 μm and 5 μm, respectively. At this magnification, each image was predominantly composed of the muscle tissue type in consideration, along with the associated connective tissues (Figures 1-6). For each query, a new instance was used, the model version was validated, and the image was manually uploaded into the web interfaces without any additional preprocessing and given the prompt: “In one sentence, please provide your best estimation of the tissue shown and the plane of sectioning used.”

The responses from each of the models were recorded and graded for accuracy. The accuracy for tissue identification and tissue sectioning was assessed separately. Only the word ‘longitudinal’ was accepted as a correct response for longitudinal images. ‘Transverse’ or ‘cross-sectional’ responses were accepted as a correct classification for a transverse image. The classification of true and false positives and true and false negatives was made individually for each tissue subtype. The correct identification of the muscle subtype presented was considered a true positive. The identification of an incorrect type of muscle was recorded as a false positive for the incorrect muscle type that the model responded with (e.g., the model identifying a skeletal muscle image as cardiac muscle will yield a false positive for cardiac muscle). A true negative for a muscle subtype was the correct identification of an image for a different subtype (e.g., correctly identifying a skeletal muscle image will award both smooth muscle and cardiac muscle one true negative for not identifying that image as one of their muscle subtypes). A false negative for a subtype was the incorrect identification of the actual muscle subtype as any other tissue. Each of these metrics is specific to a particular tissue type. An overall metric for the performance of each model across the muscle tissue types was obtained by macro-averaging the tissue-specific scores for each model. Classification of true and false positives and true and false negatives was graded individually for longitudinal and transverse sectioning. The correct identification of the type of sectioning used was considered a true positive. The identification of an incorrect plane of sectioning was recorded as a false positive for that incorrect plane and a false negative for the plane of sectioning in the image. A true negative refers to the grading of a different plane of sectioning as negative for the plane of sectioning of interest.

Model performance was evaluated by standard machine learning and artificial intelligence metrics such as accuracy, precision, recall, and F1 score for each of the LLMs. Accuracy measures the frequency with which the model correctly identifies the outcome. Precision depicts the positive predictive value of the model and is a measure of how often the model’s grading of an outcome as positive is an actual positive. Recall is a measure of the ability of the model to correctly identify all actual positive instances. Precision and recall were used to develop an aggregate score known as the F1 score. Additionally, non-parametric statistical tests, including Friedman’s and McNemar’s, evaluated the significance of the difference in correct identification of muscle subtype and planes of sectioning. A standard p-value of < 0.05 was used to calculate significance.

## Results

In the identification of muscle subtypes, Gemini 1.5 Flash correctly identified the muscle subtype in 101 images, Claude 3.5 Sonnet yielded 121 correct results, and ChatGPT-4o identified 229 images correctly. The breakdown of these results by muscle subtype, including the false positive, false negative, and true negative counts, is displayed in Table 1. Friedman’s test showed that ChatGPT-4o performed significantly better than Gemini 1.5 Flash (p<0.01) and Claude 3.5 Sonnet (p<0.01). Claude 3.5 Sonnet and Gemini 1.5 Flash were not statistically different in identifying muscle subtype (p>0.05). ChatGPT-4o produced the highest performance metrics with an accuracy of 0.860 and an F1 score of 0.772 (Table 2).

**Table 1:** Tissue identification performance statistics LLMs: Large language models

LLMs	Cardiac	Skeletal	Smooth	Average
Gemini 1.5 Flash				
True positive	46	66	22	44.7
False positive	50	68	5	41
True negative	150	132	195	1590
False negative	54	34	78	55.3
ChatGPT-4o				
True positive	82	98	49	76.3
False positive	38	14	3	18.3
True negative	162	186	197	181.7
False negative	18	2	51	23.7
Claude 3.5 Sonnet				
True positive	67	46	8	40.3
False positive	118	30	0	49.3
True negative	82	170	200	150.7
False negative	33	54	92	59.7

**Table 2:** Tissue identification performance metrics

Metric	ChatGPT-4o	Claude 3.5 Sonnet	Gemini 1.5 Flash
Accuracy	0.86	0.637	0.46
Precision	0.818	0.656	0.596
Recall	0.763	0.403	0.447
F1 Score	0.772	0.38	0.46

When identifying the plane of sectioning, McNemar’s test found all three LLMs to produce significantly different results from each other (p<0.01), with Claude 3.5 Sonnet detecting 169 correctly, ChatGPT-4o classifying 158, and Gemini 1.5 Flash identifying 107. Breakdowns by the two planes of section are included in Table 3. Claude 3.5 Sonnet demonstrated the highest performance metrics with an accuracy of 0.577 and an F1 score of 0.472, while ChatGPT-4o produced an accuracy of 0.534 and an F1 score of 0.396, and Gemini 1.5 Flash had a 0.396 accuracy and 0.344 F1 score (Table 4). 

**Table 3:** Plane of sectioning performance statistics LLMs: Large language models

LLMs	Longitudinal	Cross-Sectional	Average
Gemini 1.5 Flash			
True positive	95	12	53.5
False positive	85	54	69.5
True negative	12	95	53.5
False negative	55	138	96.5
ChatGPT-4o			
True positive	150	8	79
False positive	134	0	67
True negative	8	150	79
False negative	0	142	71
Claude 3.5 Sonnet			
True positive	150	19	84.5
False positive	117	0	585
True negative	19	150	84.5
False negative	0	131	65.5

**Table 4:** Plane of sectioning identification performance metrics

Metric	ChatGPT-4o	Claude 3.5 Sonnet	Gemini 1.5 Flash
Accuracy	0.534	0.577	0.396
Precision	0.764	0.781	0.355
Recall	0.527	0.564	0.357
F1 score	0.396	0.472	0.344

## Discussion

This study was focused on assessing the ability of the most popular multimodal LLMs to classify images of several types of muscle histology presented and identify the plane of sectioning of each image. The tissue identification and plane of sectioning abilities were assessed independently for each model. The tissue identification results depict the ability of each of the models to accurately describe the muscle histology presented in the image. The results demonstrated that of the models examined, ChatGPT-4o was most effective at identifying the muscle type presented. The plane of sectioning identification results showed that all the chosen models performed poorly when trying to identify the plane of sectioning. However, of the three models, Claude 3.5 Sonnet marginally performed the best with an F1 score of 0.472. In pathology workflows, the accurate identification of the plane of sectioning used is critical in the accurate interpretation of histological features and patterns or effective identification of sectioning artifacts [17]. The poor performance these models demonstrated in identifying the plane of sectioning represents a limitation of these untrained LLMs that must be resolved before they can be effectively integrated into clinical use.

While the exact decision-making process of the models is largely obscured due to the black-box nature of LLMs, examining the results showed some noteworthy trends. First, all the LLMs demonstrated a tendency to classify all images as one of the striated tissues, such as cardiac or skeletal muscle. This is evidenced by the higher accuracy and a higher false positive rate for the striated muscle types. In addition, Claude 3.5 Sonnet showed a particular tendency to classify the samples presented as cardiac muscle, shown by the high accuracy and 59% false positive rate for this muscle subtype. The results also showed that all three models performed poorly with smooth muscle image identification, and no model correctly identified more than 50% of the smooth muscle dataset.

Among the muscle tissue subgroups, smooth muscle was most often misclassified as non-muscle tissue, and the LLMs often misidentified these images as nervous tissue, glandular tissue, or cartilage. Each model showed similar rates of non-muscle tissue subtype misclassification, with Gemini 1.5 Flash incorrectly detecting 46, ChatGPT-4o mistyping 43, and Claude 3.5 Sonnet missing 42 images. These patterns show that while LLMs may be able to recognize features and patterns characteristic of striated muscle tissue, their lack of success in identifying features of smooth muscle represents another obstacle for diagnostic or clinical use. However, the performance of these models in identifying skeletal muscle suggests they may be valuable tools in early histology education.

The results also showed interesting trends regarding the plane of sectioning classification. Analysis of the responses regarding plane of sectioning showed that the models excelled more at identifying longitudinal tissue than at identifying cross-sectional imaging. This may be due to the distinctly identifiable and unique features present in longitudinal muscle tissue sectioning, such as intercalated discs, striations, and Z-lines. This performance disparity is illustrated clearly through the 100% recall performance that ChatGPT-4o and Claude 3.5 Sonne demonstrated for longitudinal muscle and the 5.3% and 12.7% recall performance the models demonstrated, respectively, when presented with cross-sectional muscle images. The false positive rate for longitudinal images was 57%, 89%, and 78% for Gemini 1.5 Flash, ChatGPT-4o, and Claude 3.5 Sonnet, respectively. These tendencies show that while these untrained models show immense potential, they need to be further specialized before being integrated for decision support systems or as educational tools.

As a foundational investigation on LLM performance in histology evaluation, this study presents some limitations that should be considered. First, only muscle tissue was used. As such, the generalizability of this investigation is limited, and further studies should be performed to assess model performance over a range of different tissue types. Second, only histologically normal images were used, and effectively assessing the ability of these multimodal LLMs for use in clinical practice requires the study of their performance when evaluating pathological tissue slides. Additionally, many ethical concerns arise with the use of AI, such as LLMs, in medical applications [18]. One of the main ethical concerns for LLM integration in medicine involves their 'black box' nature, referring to the opaque inner workings of these models [19-21]. Many AI tools do not have publicly available documentation, and of those that do, most models do not have methods of interpretation built in to explain the decisions that are made. This technical limitation is of particular concern in medical applications where the rationale for diagnostic decision-making is vital and carries legal and clinical consequences. As such, the lack of interpretability of the decisions made by LLMs is a technical and ethical challenge and may be a barrier towards clinical adoption [22-24].

Future revisions of these studies should focus on including confidence scoring for the LLM decisions and rationale for outputs. Moreover, public LLMs, such as the ones used here, are frequently updated and continually learn new material. It is possible that their accuracy in identifying histology slides can increase in the future or even decrease, depending on the levels/quality of training and quantity of misinformation presented. In future studies, ascertaining a confidence level associated with each response can provide evidence to help determine if models demonstrate a tendency for a default response in instances of higher uncertainty.

## Conclusions

Overall, the findings suggest that of the untrained LLMs assessed, ChatGPT-4o performs best in tissue identification and is therefore likely more suited to be a foundation for further training and use in histopathological applications. However, the results indicate that the poor performance these models showed in identifying the plane of sectioning may be a hurdle in implementation. Furthermore, extensive training on the plane of sectioning classification is essential. Nevertheless, the findings of this study suggest the notion that while these public LLMs are not yet suited for unsupervised use, they may have a role as supportive tools in digital pathology applications. They may be of value in educational settings or the support of rapid triage systems.

As opposed to conventional computerized visual classification systems, which are typically heavily trained on narrow datasets with poor generalizability, these more generic LLMs may need less specialized training and fine-tuning and could offer greater flexibility as a decision support system. Trained LLMs focused on pathological applications could be vital components of effective and accurate pathology workflows and should continue to be examined.
